# Quantifying, Understanding, and Correcting for Delays in Routine Dengue Case Reporting in the Americas

**DOI:** 10.4269/ajtmh.26-0020

**Published:** 2026-06-18

**Authors:** Katie M. Susong, Ahyoung Lim, Kishen Joshi, Joseph Clarke, Katharine Sherratt, Oliver J. Brady

**Affiliations:** ^1^ Department of Infectious Disease Epidemiology and Dynamics, Faculty of Epidemiology and Population Health, London School of Hygiene & Tropical Medicine, London, United Kingdom;; ^2^ Centre for Mathematical Modelling of Infectious Diseases, Faculty of Epidemiology and Population Health, London School of Hygiene & Tropical Medicine, London, United Kingdom

## Abstract

Dengue virus is a growing public health challenge in the Americas, where endemic–epidemic transmission and unprecedented surges demand timely and accurate surveillance. Although real-time reporting, such as the Pan American Health Organization’s (PAHO) database, is central to trend analysis, its case counts are subject to reporting delays and backfilling, which can lead to an initial underestimation of the size of an epidemic and result in insufficient control efforts. A novel, longitudinal dataset of weekly WHO PAHO dengue reports (2022–2025) was compiled, capturing successive data across 46 countries and territories. This dataset enables quantification of reporting factors across delays of up to 60 weeks. Analysis reveals that 91.4% of countries exhibit reporting delays. The average threshold to reach the final case count was 13.9 weeks (interquartile range: 3–17 weeks). Regression modeling revealed that countries that were classified as at very low risk for disaster and had a small population experienced fewer delays in reporting. When the size and severity of an outbreak are considered, severity exerts a stronger influence, resulting in fewer timely reports. The marked heterogeneity across countries in delay dynamics underscores the need to understand and correct for the causes of reporting delays on a country-by-country basis. The study findings provide methodological insights into incorporating robust priors into near-real-time forecasting when direct observations of reporting delay are unavailable. A foundation for improved modeling, enhanced outbreak response, and more resilient public health decision-making at both regional and global scales is established in the present study.

## INTRODUCTION

Dengue is an emerging global vector-borne infectious disease that places 5.66 billion people in more than 120 countries at risk of infection.^1^ In recent years, the Americas have reported the highest number of dengue cases.^2^^,^^3^ Epidemiological data from 2023 and 2024 have revealed an alarming surge in the number of dengue cases in both endemic countries, such as Brazil, and countries with historically lower transmission (e.g., Argentina and Paraguay).^4^^,^^5^ Within the Americas, the WHO and the Pan American Health Organization (PAHO) support initiatives to enhance surveillance, collaboration, and control. ^6^ Since 1980, PAHO has been aggregating dengue surveillance under a reporting scheme for all participating nations; 46 nations and territories submit the cumulative number of dengue cases, as well as incidence, the number of severe cases, the number of deaths and fatalities from dengue, and entomological surveillance data.^7^ This international database serves as the sole publicly available source of dengue case surveillance data for some countries within the region.^3^ Timely epidemiological surveillance data are essential for understanding disease patterns, identifying risk factors, and monitoring and informing long-term regional public health policy.^8^ Although the importance of timely data is clear, data are not always reported promptly.^9^ Surveillance systems, such as mandatory passive reporting systems undertaken in the PAHO region, are extensive systems that involve point-of-care testing, laboratory facilities, local and regional public health agencies, national public health agencies, and international cooperation. This complexity means that identifying cases and adding them to a multinational data system involves multiple people across multiple agencies, and the process can take anywhere from days to months.^10^ The cases initially reported are often underreported, meaning that the actual number of cases that will be detected by the surveillance system is higher than those reported, or overreported when more cases are listed than will ultimately be included in the case records. Within the surveillance system, laboratory and public health personnel can apply correction of over- or underreporting over time, culminating in a process known as backfill.

**Figure 1.**
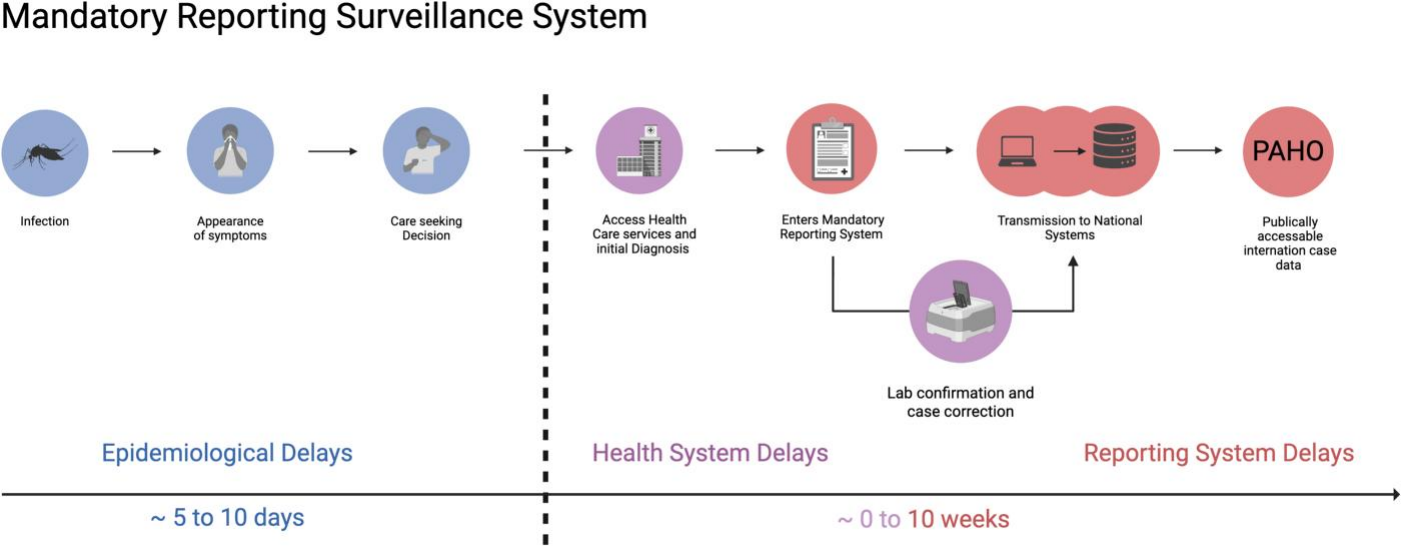
Diagram of a typical mandatory reporting system highlighting how delays in reporting occur before or after a case enters the healthcare and reporting systems. Epidemiological delays occur between the point of infection and the receipt of care, whereas postreporting delays can occur between the receipt of care and the presentation of publicly available data. Created in BioRender (2026; Toronto, Canada; https://BioRender.com/p2cuibz).

The delays encountered in infectious disease surveillance can be categorized into two stages: delays that occur before a case is reported to the disease surveillance system and delays that occur after a case has been reported (Figure 1). Delays before a case enters the system can be termed epidemiological (or prereporting) delays. Broadly, an epidemiological delay is the time between two epidemiological events. When considering dengue case data, the critical delays can be defined by the incubation period (IP), the interval between an infectious mosquito bite and symptom onset.^11^ Passive surveillance is typically positioned within points of care and only detects cases after symptom onset. For dengue, an IP of 3 to 10 days results in a 3- to 10-day delay in reporting.^12^ In addition to IP, cultural attitudes and personal decisions regarding seeking care may contribute to this initial delay.^13^ After a case has been brought to a point of care, there are additional delays that can occur related to the logistics of the surveillance system, as the detection of a case is communicated from the site of detection to regional, national, or international public health agencies. These delays are postreporting delays, which will be referred to as logistic delays.^10^ Typically, underreporting of cases during the initial reporting is followed by semiregular backfilling that addresses this underreporting as information about additional cases travels from local to international databases. These reporting delays can occur at all levels of the system: local, regional, national, and international (e.g., WHO/PAHO). The extent of this delay and the regularity of backfilling could be influenced by various socioeconomic (e.g., access to points of care, investment in public health system) and epidemiological conditions (e.g., disease incidence, severity, case fatality ratio [CFR]) that vary by disease and location and over time. The overall investment in the healthcare system, access to services, and the robustness of the infrastructure for disease reporting will affect the initial efficiency of the system. As demonstrated by experiences during the coronavirus disease 2019 pandemic, investment in and improvement of reporting infrastructure can dramatically reduce the postreporting delay.^14^ Other sources of postreporting delay could be exacerbated by the epidemiological conditions at a given time. Outbreaks larger than historic occasions could overwhelm the healthcare system, resulting in a backlog of cases at multiple time points. Overwhelmed point-of-care facilities could result in delayed reporting of suspected cases by healthcare providers. Delays in confirming these cases by inundated regional and national testing laboratories could further postpone confirmation of case status and delay stabilization of case counts without further backfilling. If there is an increase in the severity of the outbreak, exhausted staff at public health departments may fall behind in reporting, further contributing to the delay in final case counts.

Epidemiological and logistical delays are present in a wide range of surveillance systems across many infectious diseases.^15^ The presence of these delays can hamper real-time situational awareness and response.^16^ The need to consider these delays in case data has driven development in nowcasting and data delay models. Nowcasting methods often account for both epidemiological and logistical delays.

Epidemiological delays can be estimated from a delay distribution that is specific and standard to a pathogen, but it requires individual case tracking for direct calculation. The logistical delays of a surveillance system, such as the WHO/PAHO system, can be observed and described if the data are preserved longitudinally. To understand how logistical delays are presented and described within longitudinally maintained case data, consider this example: for epidemiological week (EW) 20, dengue cases are reported in EWs 21, 25, and 30 of that year and in a review of the season the next year. These case counts are 90, 95, 100, and 100, respectively. In this example, there are case counts with reporting delays of 1, 5, and 10 weeks, along with what can be considered the final reported case count. To begin to evaluate the hypothetical surveillance system, the reporting completeness at each reporting delay will be calculated. Reporting completeness is the proportion of cases reported at a given reporting delay, relative to the final number of cases reported (also termed the validation ratio).^9^ In this example, the reporting completeness at a delay of 1 week was 0.9, meaning that 90% of the total cases will be included in the surveillance system 1 week after they enter the system. For the next delay (5 weeks), reporting completeness was 0.95, and at a 10-week delay, it was 1. Although useful, in data delay models and the nowcasting literature, reporting completeness is frequently transformed into a scaling factor, which is termed a reporting factor. The reporting factor, which is the inverse of reporting completeness, can be used to rescale real-time cases. Using this measure, in the study example, with a reporting delay of 1 week, the reporting factor is 1.1, which provides the scaling factor to correct the 90 observed cases to 100 final cases. Many nowcasting methods require an estimate of the reporting factor.^9^^,^^11^^,^^17^ There are multiple strategies for estimating the reporting factor that rely on historic or real-time data, which include the corrections made during backfill. Considering either reporting completeness or the reporting factor, the time to achieve a value of 1 is the delay required to receive the most accurate case count. The time can be summarized across all the weeks or in the same week across multiple years to determine the average time to final case counts.

Using geographically extensive dengue data from the collection of international case data by PAHO, it is possible to assess how spatial and epidemiological characteristics affect the reporting factor throughout a season. Although work has been performed to establish broadly applicable methodologies for incorporating reporting factors into disease nowcasting and forecasting models, understanding the variation in these reporting factors often lies beyond the scope of such efforts. Typically, extensive historical data have been used to estimate reporting factors.^9^ These data are usually temporally comprehensive but geographically limited. Consequently, little progress has been made in determining how the key drivers of reporting factors vary across geographic regions. Moreover, limited efforts have been made to understand the impact of changing outbreak conditions, such as size, severity, and duration, on reporting factors. Analyzing reporting delays requires capturing snapshots of the regularly updated databases over time. Many databases do not preserve previous versions of case data, instead maintaining only the most accurate version. To the best of the authors’ knowledge, the study collection of dengue records is the first time that dengue case data have been preserved in this way. This lack of historical data (or strongly predictive proxies) makes understanding the underlying mechanism that contributes to reporting factors more critical. Here, reporting delay factors for 46 countries across PAHO are characterized, and insights into the drivers of reporting delay magnitude that could generalize to other settings to improve outbreak situational awareness, early warning, and response are given.

## MATERIALS AND METHODS

### Longitudinal dengue case surveillance data.

In the Americas, the WHO/PAHO has published national dengue epidemiological surveillance data since 1980. Currently, 46 countries and territories systematically report the weekly total number of cases, incidence, number of severe cases, number of deaths, and dengue fatality rate, as well as additional entomological surveillance data. This database is updated semiregularly. Case counts are backfilled without maintaining a publicly available record of the change. Further information and access to the WHO/PAHO dengue database can be found at https://www.paho.org/en/arbo-portal/dengue-data-and-analysis.^7^ To track these backfilling events weekly, on Monday from June 13, 2022 to February 6, 2023 (weeks = 45), the WHO/PAHO dengue surveillance database was manually downloaded and saved. In July 2024 (July 26, 2024), the WHO/PAHO dashboard was revisited, and data for 2022/2023 were downloaded manually. This version was considered the final case counts for 2022/2023. To further enhance data collection and support the ongoing development of this data resource, an automated process was developed to routinely download and store the WHO/PAHO dengue surveillance database. This automated process was initiated on June 19, 2024. The complete database is downloaded and stored daily at ∼10:00 am. The WHO/PAHO continued to improve and update the web application, which led to brief periods when the automated system failed, resulting in small gaps in the data records. These gaps in data availability are smaller than the observed update schedule. Within the study analysis, 2024/2025 real-time data were limited to the weeks from June 19, 2024 to May 18, 2025. This allowed for the use of data (September 3, 2025) with a minimum of 20 weeks for correction as final count data. A minimum of 20 weeks was selected because, during 2022/2023, after 20 weeks, the percentage of cases that were not stabilized to the number seen in the final case counts was less than 5%. This resulted in two seasons, 2022/2023 and 2024/2025, which were available for analysis. This novel data resource for dengue forecasting continues to be collected; further information and the data can be found at https://github.com/DengueGlobalObservatory/PAHO-crawler.

### Quantifying delays in reporting.

The delay in reporting was expressed in weeks and defined as the difference between the EW assigned to the case and the epidemiological week the data were downloaded, termed the reporting week (rEW). For instance, in rEW 35, cases that occurred in EW 35 will have a delay of 0 weeks, whereas cases that occurred in EW 30 have a delay of 5 weeks.

Here, the impact of this delay on case counts is expressed as a reporting factor that was calculated for each observation using the following equation:

$$rf_{ts}\left(d\right)=\frac{F_{ts}}{N_{ts}\left(d\right)}$$

where rf is the reporting factor; N_ts_(d) is the case count for the given year (s) and EW (t) at a given delay (d); and Fts is the final case count for the same year and EW.^9^

For each PAHO country, the threshold reporting delay was determined. The threshold reporting delay was defined as the minimum delay (in weeks) at which the mean reporting factor equals 1 (±0.01). After the threshold reporting delay, the number of reported cases and the final case count would be expected to be equal. In addition, the mean minimum delay for the reporting factor is calculated to equal 1 (±0.01) for a single observation. This is a similar value to the threshold reporting delay; however, the threshold reporting delay introduces uncertainty in the reporting factor at each delay, whereas the mean minimum delay introduces the uncertainty of the time to a reporting factor of 1.

### Seasonal and spatial variation in reporting.

To begin to understand how reporting varied during the season and across countries, weekly reporting factor values were averaged over the two seasons to produce a unified seasonal profile per country. A 4-week rolling mean and SD of the reporting factor were computed for each country to capture short-term trends.

To investigate the observed heterogeneity between countries and across the season, the analysis of reported cases with specific delays was compared with the final cases using a generalized linear regression framework that incorporated spatial and temporal variables. A quasi-Poisson distribution was selected after evaluating model fit for overdispersion, collinearity, and heteroscedasticity. Both reported cases and the duration of reporting delays were included in all regression analyses. Predictive performance was assessed using 5-fold cross-validation, repeated 10 times, with a different random partitioning of the data into folds for each repetition. The root mean square error (RMSE), mean absolute error (MAE), and deviance metrics were calculated for each regression. These metrics were expressed as incidence rates to account for differences in population size and, consequently, the case counts between countries. Regressions incorporating a single spatial or temporal covariate were analyzed to assess the independent value of each metric. Model performance was ranked by RMSE (reported as an incidence). The best-performing solo spatial and temporal variables without colinearity were integrated into “complex” models (i.e., models with multiple covariates of interest; Table 1), and the predictive capacities of these models were compared. Finally, given that Brazil accounts for 82.1% of all cases reported in the Americas during this time frame, the temporal analyses were repeated, excluding Brazil as a sensitivity analysis.

**Table 1:** Quasi-Poisson generalized linear regression framework used to assess whether the variation in delay across countries can be determined using broadly available conditions of the country or epidemiological conditions of the dengue season

Model ID	Formula
baseline	Nts_V ∼ Nts:d
Simple spatial models	
Country	Nts_V ∼ Nts : (d + Country)
region	Nts_V ∼ Nts : (d + ID)
GDPc	Nts_V ∼ Nts : (d + GDPc)
Inform	Nts_V ∼ Nts : (d + imform_risk)
Inform_class	Nts_V ∼ Nts : (d + risk_class)
Inform_epi	Nts_V ∼ Nts : (d + epidemic)
timeH	Nts_V ∼ Nts : (d + Pop_Weighted_Travel)
Simple temporal models	
EW	Nts_V ∼ Nts : (d + EW)
holiday	Nts_V ∼ Nts : (d + holiday)
Incidence	Nts_V ∼ Nts : (d + incidence)
Incidence_diff	Nts_V ∼ Nts : (d + diff_incidence)
Disease_sev	Nts_V ∼ Nts : (d + sev_incid)
CFR	Nts_V ∼ Nts : (d + CFRts)
Complex models	
spatial_complex	Nts_V ∼ Nts : (d + risk_class + Pop_Weighted_Travel)
epi_complex	Nts_V ∼ Nts : (d + CFRts + sev_incid)
Complex	Nts_V ∼ Nts : (d + risk_class + Pop_Weighted_Travel + CFRts + sev_incid)

CFR = case fatality ratio; EW = epidemiological week; GDPc = gross domestic product per capita.

Seven spatial covariates and six temporal variables were considered to evaluate variation in reporting delays across countries. The spatial covariates include 1) region, 2) country, 3) gross domestic product per capita (GDPc), 4) index for risk management (INFORM) class (The Joint Research Centre of the European Commission, Brussels, Belgium), 5) INFORM score, 6) INFORM epidemics score, and 7) the population weights mean time to healthcare. Country and region were included because the authors hypothesized that there would be high variability between countries and regions that might not be captured by any other selected variable. The GDPc was included because it has been found to be linked to the extent and burden of epidemics and pandemics.^18^^,^^19^ The GDPc was identified via the World Bank (Washington, DC, USA).^20^

Preparedness and risk are highly complex, multifaceted concepts that can be influenced by a wide array of factors. To evaluate the impact of a country’s preparedness efforts and vulnerability on surveillance outcomes, the INFORM metric was leveraged. The Joint Research Centre of the European Commission leads a multistakeholder initiative that has developed a quantitative analysis product to support and inform decision-making concerning humanitarian crises and disasters (https://drmkc.jrc.ec.europa.eu/inform-index). These products include the INFORM risk index, an open-source risk assessment tool aimed at prevention, preparedness, and response to humanitarian crises and disasters.^21^ The INFORM risk index integrates 80 distinct indicators to assess the hazards present, the vulnerability of exposed populations, and the available resources to respond to and manage these hazards. For the study analysis, three components of the INFORM risk index were taken into consideration: 1) the overall risk score (0–10, with increasing risk), 2) the risk classification (ranging from very low to very high), and 3) the epidemic-specific risk score (0–10, with increasing risk).

Finally, the authors considered how access to points of care, as the point where cases enter the surveillance system, could affect the downstream reporting system. The Malaria Atlas Project (https://malariaatlas.org/) developed a 1-km scale mapping product of time to health care (https://malariaatlas.org/project-resources/accessibility-to-healthcare).^22^ In conjunction with the Worldpop (https://www.worldpop.org/) 1-km scale population data,^23^ this provided the population-weighted average time to health care services by country.

The temporal covariates included 1) EW, 2) public holidays, 3) incidence, 4) the difference from historic incidence, 5) disease severity, and 6) the relative CFR (Table 1). In daily infectious disease monitoring, surges in cases are often observed after weekends due to a backlog in data entry.^24^ Public holidays further exacerbate these short-term changes in reporting delays.^25^ To consider the effects of public holidays on reporting factors, weeks were defined as including a public holiday or not. For the entire region, two holiday periods were considered: 1) the Christmas/New Year’s season (epidemiological week range [EWr] 50–1) and 2) carnival (2023: EWr 7–8; 2025: EWr 9–10). Furthermore, the evaluation of changes in the reporting factor at a given delay over time suggested that certain country-specific holidays might drive its trends. These holidays included 1) Día de la Revolucion (Cuba EWr = 30), and 2) Onafhankelijkheidsdag, or Independence Day (November 25; Suriname, EWr = 47).

The authors also hypothesized that the temporal variation in the reporting factor at a given delay could be driven by the epidemiological characteristics of the dengue outbreak at that time. The authors considered that an increase in the incidence or severity of cases compared with previous years could inform the performance of dengue surveillance systems and, consequently, affect the reporting factor at a given delay. The mean historical weekly incidence and CFR were calculated using PAHO dengue data (2014–2020). Using these historical values, the 1) change in incidence and 2) relative case fatality rate were calculated and considered in the study analysis. Additionally, the proportion of severe cases was considered in the study analysis.

## RESULTS

**Figure 2.**
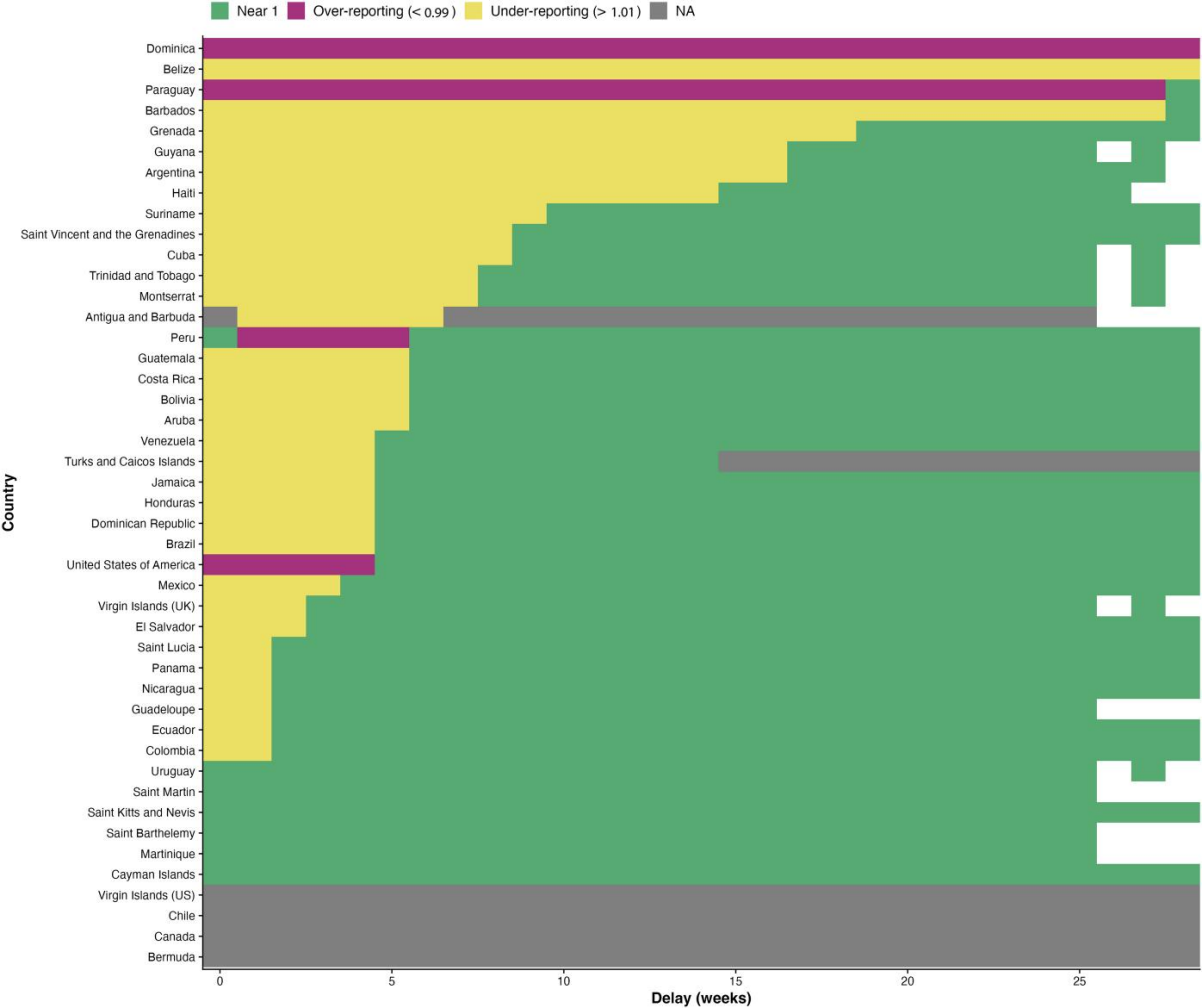
Average reporting factor by delay (in weeks) for all Pan American Health Organization Nations across the 2022–2023 and 2024–2025 seasons. Reporting factors near 1 (0.99–1.01) indicate that the cases reported at that delay have coalesced to the final cases reported. Underreporting occurred when the reporting factor was more than 1.01, whereas overreporting occurred when it was less than 0.99.

In the PAHO region, most (91.4%) countries exhibit some form of delay in reporting accurate dengue case figures (Figure 2). Most of these delays (78.6%) presented as underreporting of cases, whereas a small number of countries exhibited overreporting (7%). The time taken to resolve under- and overreporting varied, but on average, accurate numbers were reported within 13.9 weeks (interquartile range: 3–17 weeks; Supplemental Table 1). The pattern in reporting delay varied notably by country. Central American Isthmus (including Mexico) and non-Latin Caribbean countries experienced higher initial delay than countries in other regions (Supplemental Figure 1).

**Figure 3.**
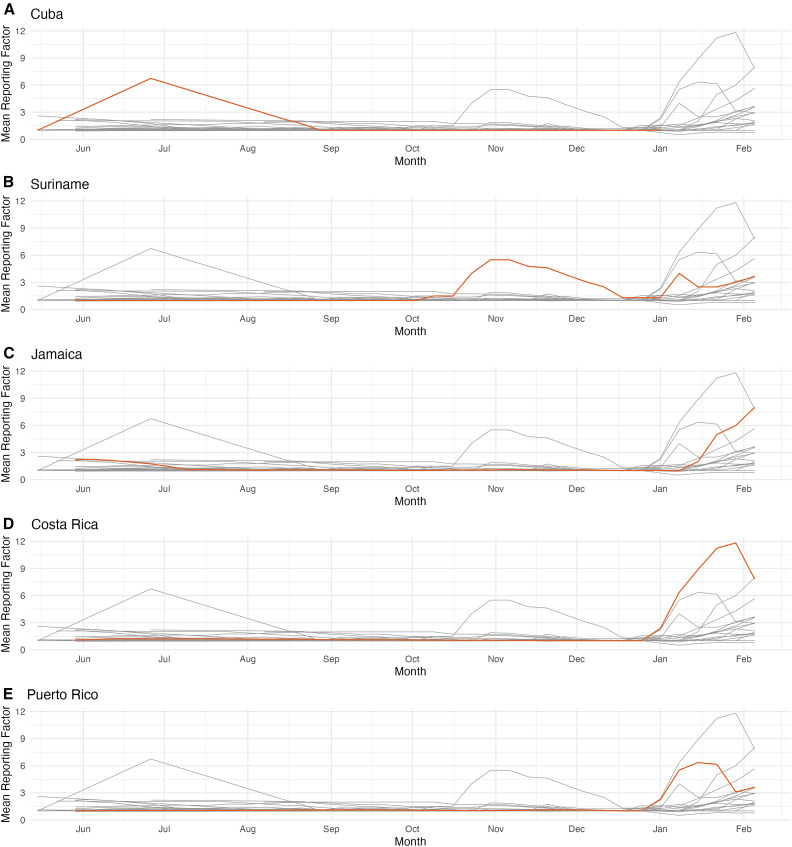
Four-week rolling average of reporting delay, irrespective of the delay for Pan American Health Organization nations. National data with distinctive trends are highlighted across the panels. (**A**) Cuba is the sole country to exhibit an increase in underreporting in early summer. (**B**) Suriname exhibits a unique peak in the fall. (**C–E**) Jamaica, Costa Rica, and Puerto Rico are the best examples of a broader trend of peaks around Christmas, New Year’s, and Carnival.

In addition to varying by country, specific weeks displayed larger reporting factors across all delay lengths. Three peaks can be seen when the moving average (4 weeks) is visualized across seasons by country (Figure 3). The first occurred in July, the second occurred in November, and the third occurred in December/January. The increase in the reporting factor in July 2022 was almost entirely driven by Cuba, possibly because of the closure of public offices or delayed treatment-seeking around Día de la Rebeldía Nacional (Independence Day; July 26). The second increase in the reporting factor was observed only in Suriname and is likely due to the closure of public offices on Onafhankelijkheidsdag (Independence Day; November 25). The rise in December/January is likely attributed to the closure of public offices during the Christmas and New Year’s holidays. The increase in the reporting factor is not uniform across all countries and was primarily influenced by Costa Rica, Puerto Rico, and Jamaica; however, most nations exhibit some increase in the reporting factor during that time. Cuba and Costa Rica rely heavily on government-run and centralized reporting infrastructure. Additionally, these are nations where national holidays may have a more uniform and easily observable impact on the entire country’s surveillance system than in a large, geographically diverse country.

### Explaining the variation in the reporting factor.

Given the large variation in the reporting factor (i.e., the relationship between observed and final cases), regression models were fitted to identify common patterns driving these relationships. Models were designed to consider the final case count as a response of the observed cases, reporting delay, and other specified covariates (Table 1). The baseline model, including the delay length and observed cases, resulted in the poorest performance metrics.

**Figure 4.**
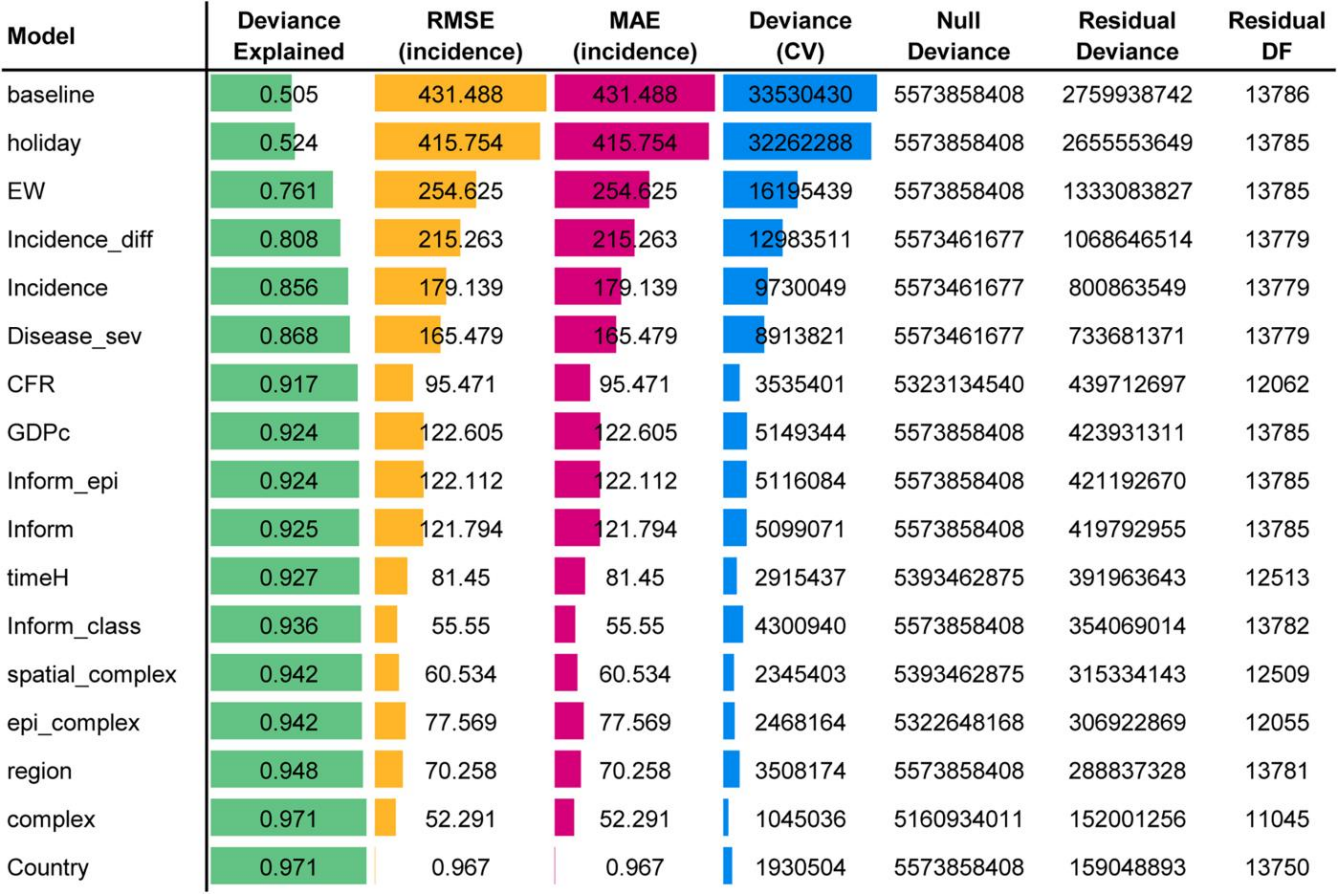
Metrics of fitness for quasi-Poisson generalized linear regressions. The metrics involved include deviance explained, null deviance, residual deviance, and residual degrees of freedom. In addition, the results of k-fold cross-validation (k = 5, repeats = 10) are reported as the root mean square error (RMSE; incidence), mean absolute error (MAE; incidence), and deviance. For deviance explained, RMSE (incidence), MAE (incidence), and deviance colored bars correspond to the values.

All single-variable spatial models outperformed the baseline model (Figure 4). The relative performance of these models, in decreasing order, was INFORM class, region, population-weighted time to healthcare, INFORM score, INFORM epidemic score, and GDPc (Figure 4). INFORM class and region are both categorical variables that may define underlying similarities between counties because of their spatial proximity or underlying policy decisions. The INFORM class defines each country as being at very low, low, medium, high, or very high risk of disaster. In the study analysis, decreasing risk reduced the difference between the reported and final cases across all delays (Supplemental Table 2).

Given the potential influence of temporal variation on the reporting factor, a set of temporal single-variable regression models was evaluated. All temporal models outperformed the baseline model. Based on population-adjusted RMSE, the best-performing temporal covariate was the CFR. This was the only covariate to outperform any of the spatial models, surpassing the INFORM score, INFORM epidemic score, and GDPc. The other temporal models, in descending order of performance, were as follows: relative disease severity, current incidence, deviation from historical incidence, EW, and public holidays. Covariates that reflect the severity of the disease burden had a greater negative impact on the reported cases than variables that indicated the size of the outbreak (Figure 4 and Supplemental Table 2). When the country variable was added to the baseline model, the model outperformed all single-variable models (Figure 4 and Supplemental Table 3).

Finally, to determine if multivariate spatial and temporal covariates could compete with the country model, three complex models were created that included the best-performing variables from the single-variable models, without collinearity. The complex models were defined as a complex spatial model, a complex temporal model, and a complex combined model (Table 1). All three models were unable to outperform the country model (Figure 4). However, the combined complex model performed exceptionally well. The RMSE and MAE are only slightly higher than those of the country model, and there is an improvement in deviance (Figure 4). Although a model explicitly defined by country can be used to evaluate real-time data from that country, it does not provide a broader understanding that can be applied to countries with limited or no historical delay data. The comparable performance of the combined complex model indicates that even when historical data are lacking, some aspects of the likely delay dynamics in that country can still be inferred.

### Considering Brazil: sensitivity analysis.

The large population and dengue outbreaks in Brazil may have had an undue influence on the performance of the various spatial and temporal covariates. To assess the influence of Brazil on model performance, a sensitivity analysis was conducted, excluding Brazil from model development and evaluation. The exclusion of Brazil resulted in a decrease in the absolute performance of models and a change in their relative performance. Incidence and the difference from the average historical incidence did not outperform the baseline model. The best-performing complex model included only spatial variables. The country model was still the best-performing model overall (Supplemental Figure 2). The large size of outbreaks in Brazil, compared with the rest of the region, affects the broader results but does not change the general conclusions derived from the present work.

## DISCUSSION

The global response to the rising risk of dengue includes expanding collaborative, real-time surveillance of cases from subnational to international levels.^26^ International commitment to these efforts has led to a growing wealth of real-time data,^7^ critically important for early outbreak detection and containment, resource allocation, the swift implementation of public health measures, real-time communication with the public, and the coordination of global responses.^27^ Even the most efficient real-time reporting systems are not instantaneous. Reported cases can take days to weeks to accurately reflect the true count of cases at a given time point.^10^ This lag in information can impact the timing and effectiveness of a variety of public health interventions.^16^ Reporting, such as from the WHO/PAHO database, provides critical information for public health, yet these data are subject to reporting delays. Here, the authors show that underreporting of dengue cases is widespread across PAHO countries over extended periods, but that these delays follow predictable patterns. Adjusting for these delays could improve real-time situational awareness and the timeliness of outbreak response.

The study analysis reveals that the processes underlying reporting delays are highly heterogeneous between countries. A total of 63% of countries experience underreporting that is resolved within 10 weeks or less. Even a few days of prereporting delay are known to significantly affect disease control in vector-borne diseases like malaria and dengue.^28^ Given dengue’s short generation time,^12^ delays of several weeks in postreporting (i.e., data becoming available for action) could have an even greater operational impact on outbreak response and control efforts.^29^

A total of 6.9% of countries experience underreporting that fails to resolve during the observed season and is only corrected in the final data. The authors propose that delays of this magnitude are not due to infrastructural inefficiencies or logistical limitations but are instead a consequence of systemic choices that may limit updates during a specific time period. This could be due to the prioritization of limited personnel for other pressing public health concerns or economic incentives to delay the reporting of significant outbreaks.

In a few countries (10.8%), overreporting was observed. Overreporting is typically attributed to the removal of assigned cases after they fail laboratory confirmation. This may be what occurred in Peru. However, in other countries with overreporting that did not resolve within the observed season, the mechanism is unclear. Although most nations initially exhibit some reporting errors, a few do not, indicating no over- or underreporting. These are typically small nations with a low incidence of cases (e.g., Turks and Caicos Islands, Saint Martin, Saint Kitts and Nevis, and Cayman Islands). It seems probable that the small size of the nations and the relatively low number of cases reduce the amount of postreporting delay in these nations.

Although the characterization of delay and reporting factors in the PAHO data is useful for the region, at the time of publication, the authors are unaware of any dengue surveillance system that maintains publicly available records of backfilling and updated case counts. The data used within this publication begin to address this critical gap in available data. As such, insight into the drivers of reporting factors and delays generated here may provide generalized information that can improve understanding of reporting systems, outbreak situational awareness, and early warning and response across a wider scale where more direct data are unavailable. In the authors’ exploration of the relationship between reported and final cases, they considered a variety of spatial and temporal variables that described general or public health conditions within a country (spatial variables) or the weekly dengue situation (temporal variables).

Spatial variables provided more information to the models than temporal variables. Notably, INFORM provided the greatest insight of any variable. INFORM is an open-source quantitative index of a country’s preparedness for humanitarian crises and disasters, including epidemics. For temporal variables, those reflecting real-time disease burden and severity outperformed the others, indicating that outbreak severity impacts the timeliness of the reporting system. In contrast, the relationship between reported and final cases was less sensitive to variables that indicate a larger overall outbreak size. This suggests that surveillance systems are more resilient to larger outbreaks than to increasing severity of outbreaks. Increasing severity could be expected to result in increased burdens and demands per patient on healthcare workers at hospitals and points of care. These increased demands on healthcare workers could explain why the increase in delay is more notable with increasing severity than with increasing size. When considered in a multivariate analysis, the combination of both spatial variables (INFORM risk class and population-weighted mean travel time to health care) and temporal variables (CFR and relative disease severity) yielded the best results of all covariate combinations tested.

The substantial variation in reporting delays across countries indicates a critical gap in the available knowledge. As previously stated, the data needed to directly estimate reporting factors for dengue case counts are not readily available in many countries because of current data reporting practices. Until changes in standard reporting practices are widely adopted, the authors have tested several near-universal data sources that can serve as useful priors within a Bayesian framework for estimating reporting factors in countries without data.

Annual forecasting challenges for various infectious diseases, including dengue, are organized by the Centers for Disease Control and Prevention and other agencies.^30^^–^^34^ Submissions to these challenges must address the complexities of epidemiological and postreporting delays in surveillance. For dengue, the epidemiological delay (3–10 days) and the postreporting delays (∼13 weeks) overlap. The understanding provided here offers essential information to develop methods within this forecast to specifically and accurately account for postreporting delays. This will not only enhance the accuracy of this forecast but will also enable separate consideration of the epidemiological delays.

### Limitations.

The results and conclusions presented here are limited by the novelty of the data source and the source itself. With only two seasons of data, the authors have a limited ability to assess the extent of interseasonal variability and cannot conclusively state that all of the study findings will remain valid as more data become available. The authors’ collection of this data continues; consequently, they will be able to revisit these topics with even greater certainty over time. The PAHO dengue dataset is a publicly available dataset distinct from national data maintained by public health agencies. As such, the authors are unable to confirm if these delays are accumulating within the national reporting system or only in the transition from the internal national system to the publicly available dataset. Given the current understanding of passive reporting systems, it is reasonable to assume that delays are accumulating throughout the process, but the exact attribution of these delays will require further collaboration with national public health agencies.

## CONCLUSION

The consideration of backfilling and reporting delays requires both developments in data availability, through improvements in surveillance and reporting systems, and the innovative use of data. The PAHO dengue dataset used here not only fills a critical gap in publicly available information on dengue case backfilling but also provides an unprecedented comparative view of surveillance system performance across PAHO countries. The marked heterogeneity in reporting delays across countries underscores the need to tailor surveillance enhancements to country-specific operational realities. The consideration of spatial and temporal predictors provides valuable methodological insight into constructing strong priors to guide nowcasting in situations where direct reporting delay data are unavailable. Here, the authors lay the groundwork for the future development of nowcasting models capable of explicitly disentangling and accounting for postreporting delays, thereby improving outbreak situational awareness and the timeliness of public health responses at both regional and global scales.

## Supplemental Materials

10.4269/ajtmh.26-0020 Supplemental Materials
