# Supplementary material for: Quantifying, Understanding, and Correcting for Delays in Routine Dengue Case Reporting in the Americas
Source: Am J Trop Med Hyg. 2026 Jun 18;115(2):348–57. doi: 10.4269/ajtmh.26-0020 (PMC13447814; doi:10.4269/ajtmh.26-0020)
Supplement: Supplementary file 1 [file tpmd260020.SD1.pdf]

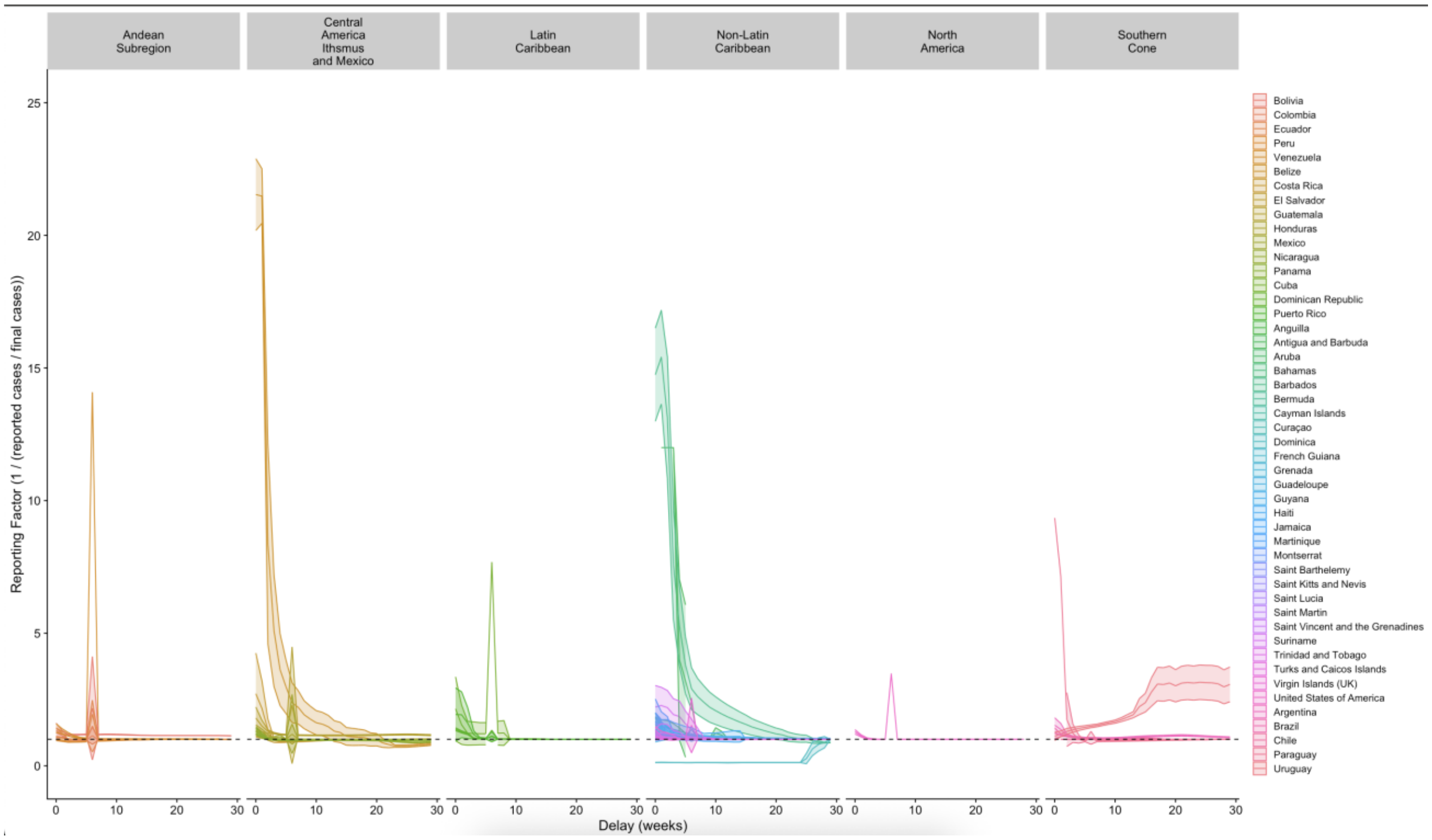

Supp Fig 1. The reporting factor of PAHO nations across delay time periods shown by regions.

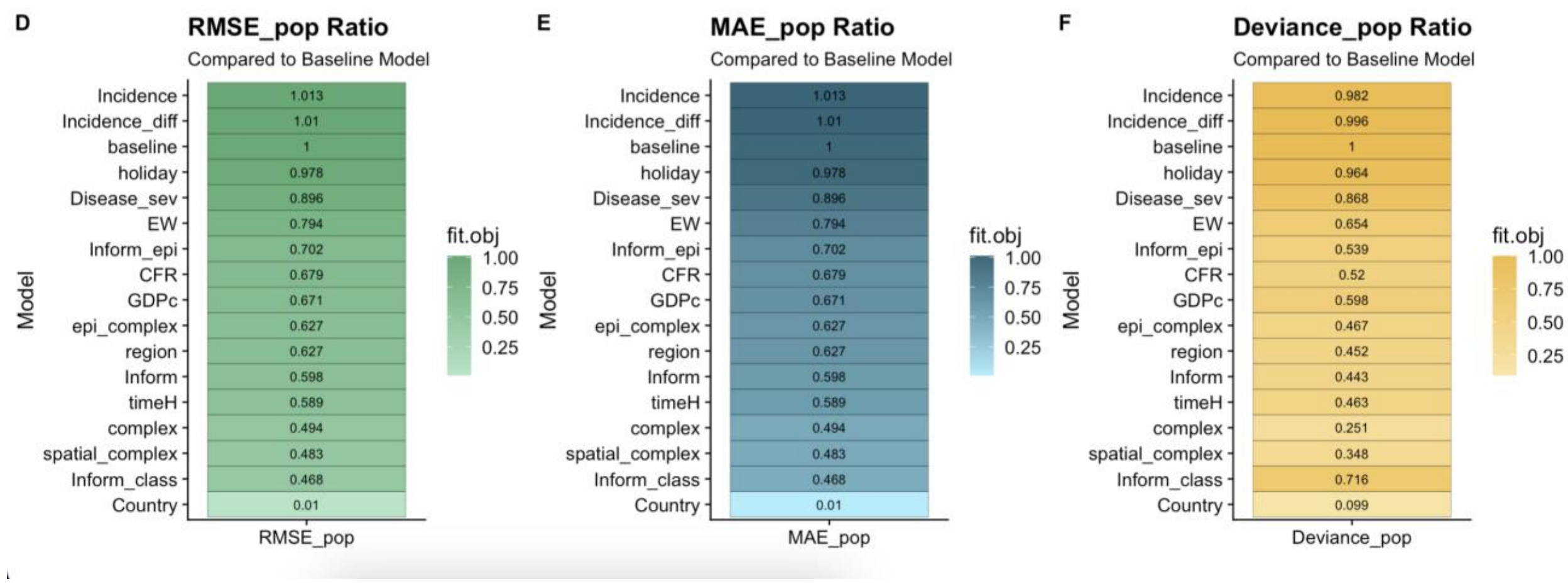

Supp Figure 2. Cross-validation metric of the sensitivity analysis where Brazil was excluded from model development and analysis.

Supplemental Material

Country	Threshold reporting delay	Threshold Reporting Factor			Count
		Mean	*Lower 95% CI*	*Upper 95% CI*	
Cayman Islands	0	1.000	1.000	1.000	23
Saint Barthelemy	0	1.005	0.995	1.015	14
Saint Kitts and Nevis	0	1.000	1.000	1.000	23
Turks and Caicos Islands	0	1.000	NA	NA	1
Chile	1	1.000	1.000	1.000	4
Nicaragua	2	1.004	0.983	1.026	63
French Guiana	2	1.000	1.000	1.000	24
Guadeloupe	2	1.005	0.998	1.013	51
Martinique	2	1.003	0.999	1.008	49
Saint Lucia	2	1.000	1.000	1.000	169
Saint Martin	2	1.000	1.000	1.000	18
United States of America	3	1.007	0.993	1.020	29
El Salvador	3	1.003	1.001	1.005	209
Ecuador	3	1.005	1.002	1.007	207
Paraguay	4	1.005	0.854	1.155	305
Anguilla	4	1.000	1.000	1.000	7
Virgin Islands (UK)	4	1.005	0.998	1.011	20
Honduras	5	1.006	1.001	1.010	289
Trinidad and Tobago	5	1.007	0.996	1.018	97
Bermuda	6	1.000	1.000	1.000	3
Jamaica	6	1.000	1.000	1.001	280
Mexico	7	1.008	1.006	1.011	285
Puerto Rico	7	1.007	1.005	1.008	270
Montserrat	8	1.000	1.000	1.000	102
Cuba	9	1.000	1.000	1.000	21
Aruba	9	1.005	1.002	1.008	50
Suriname	11	1.007	1.000	1.013	61
Saint Vincent and the Grenadines	12	1.008	0.997	1.020	81
Antigua and Barbuda	13	1.008	1.002	1.014	68

Cura√ßao	14	1.000	1.000	1.000	4
Colombia	15	0.994	0.990	0.998	283
Dominican Republic	15	1.007	1.002	1.011	276
Haiti	15	1.000	1.000	1.000	11
Costa Rica	16	0.994	0.992	0.996	282
Brazil	16	1.008	1.008	1.009	282
Peru	17	0.992	0.990	0.993	281
Grenada	17	1.007	1.000	1.014	280
Belize	19	0.994	0.745	1.244	260
Venezuela	24	1.008	1.003	1.014	10
Bahamas	25	1.000	1.000	1.000	2
Dominica	29	1.000	1.000	1.000	7
Guyana	37	1.009	1.007	1.010	269
Panama	43	0.992	0.990	0.993	269
Guatemala	46	1.002	0.999	1.005	269
Barbados	50	0.995	0.992	0.999	269
Uruguay	60	1.000	1.000	1.000	230
Bolivia	66	1.009	1.004	1.013	285

Supp Table 1. The Threshold delay for the reporting factor is set to equal 1 across PAHO nations. Include the 95% CI of the reporting factor and the number of observations in the data.

model.id	Variable	estimate	std.error	p.value	interpretation
baseline	Nts:d	0	0	0	No clear effect
region	Nts:d	0	0	0	No clear effect
	Nts:ID2	0	0	0	Positive: in Nts:ID2, early reports underestimate final cases more strongly
	Nts:ID3	0	0	0	Positive: in Nts:ID3, early reports underestimate final cases more strongly
	Nts:ID4	0	0	0	No clear effect
	Nts:ID5	0	0	0.2961	Negative: in Nts:ID5, early reports are closer to final counts
	Nts:ID6	-0.0006	0.0001	0	Negative: in Nts:ID6, early reports are closer to final counts
GDPc	Nts:d	0	0	0	No clear effect
	Nts:GDPc	0	0	0	No clear effect
Inform	Nts:d	0	0	0	No clear effect
	Nts:imform_risk	0	0	0	No clear effect
Inform_class	Nts:d	0	0.0019	1	No clear effect
	Nts:risk_classLow	-0.0033	605.7501	1	Negative: in Nts:risk_classLow, early reports are closer to final counts
	Nts:risk_classMedium	0	0.0252	0.9999	No clear effect
	Nts:risk_classVery High	-0.0005	179.4621	1	Negative: in Nts:risk_classVery High, early reports are closer to final counts
	Nts:risk_classVery Low	-2.3283	517373.135	1	Negative: in Nts:risk_classVery Low, early reports are closer to final counts
Inform_epi	Nts:d	0	0	0	No clear effect
	Nts:epidemic	0	0	0	No clear effect
timeH	Nts:d	0	0	0	No clear effect

	Nts:Pop_Weighted_Travel	0	0	0	No clear effect
EW	Nts:d	0	0	0	No clear effect
	Nts:EW	0	0	0	No clear effect
holiday	Nts:d	0	0	0	No clear effect
	Nts:holidayTRUE	0	0	0	No clear effect
Incidence	Nts:d	0	0	0	No clear effect
	Nts:incidence	0	0	0	No clear effect
Incidence_diff	Nts:d	0	0	0	No clear effect
	Nts:diff_incidence	0	0	0	No clear effect
Disease_sev	Nts:d	0	0	0.7133	No clear effect
	Nts:sev_incid	0.0004	0	0	Positive: in Nts:sev_incid, early reports underestimate final cases more strongly
CFR	Nts:d	0	0	0	No clear effect
	Nts:CFRts	0.0001	0	0	Positive: in Nts:CFRts, early reports underestimate final cases more strongly
spatial_complex	Nts:d	0	0.002	1	No clear effect
	Nts:risk_classLow	-0.0018	498.0911	1	Negative: in Nts:risk_classLow, early reports are closer to final counts
	Nts:risk_classMedium	0	1.1924	1	Negative: in Nts:risk_classMedium, early reports are closer to final counts
	Nts:risk_classVery High	-0.0005	187.5051	1	Negative: in Nts:risk_classVery High, early reports are closer to final counts
	Nts:risk_classVery Low	-2.1894	541093.594	1	Negative: in Nts:risk_classVery Low, early reports are closer to final counts
	Nts:Pop_Weighted_Travel	0	0.0028	1	No clear effect
epi_complex	Nts:d	0	0	0	No clear effect

	Nts:CFRts	0.0001	0	0	Positive: in Nts:CFRts, early reports underestimate final cases more strongly
	Nts:sev_incid	-0.0005	0	0	Negative: in Nts:sev_incid, early reports are closer to final counts
complex	Nts:d	0	0.0023	1	No clear effect
	Nts:risk_classLow	-0.0011	351.1404	1	Negative: in Nts:risk_classLow, early reports are closer to final counts
	Nts:risk_classMedium	0	1.288	1	Negative: in Nts:risk_classMedium, early reports are closer to final counts
	Nts:risk_classVery High	-0.0004	183.7673	1	Negative: in Nts:risk_classVery High, early reports are closer to final counts
	Nts:risk_classVery Low	-1.9456	597993.203	1	Negative: in Nts:risk_classVery Low, early reports are closer to final counts
	Nts:Pop_Weighted_Travel	0	0.0031	1	No clear effect
	Nts:CFRts	0	7.2751	1	Positive: in Nts:CFRts, early reports underestimate final cases more strongly
	Nts:sev_incid	-0.0005	22.1942	1	Negative: in Nts:sev_incid, early reports are closer to final counts

Supp. Table 2. Coefficients of all models except country model with a generalised linear regression framework using a quasi-Poisson distribution. See Table 1 for full structure of each model.

Country	Estimate	std.error	interpretation
Aruba	-1.871	211142.145	Negative: in Aruba, early reports are closer to final counts
Bolivia	0	2.244	No clear effect
Brazil	0	0.004	No clear effect
Cayman Islands	-4.701	789789.664	Negative: in Cayman Islands, early reports are closer to final counts
Colombia	0	0.272	No clear effect
Costa Rica	0	7.163	No clear effect
Cuba	-0.001	18.907	Negative: in Cuba, early reports are closer to final counts
Dominican Republic	0	5.59	No clear effect
Ecuador	0	1.548	No clear effect
El Salvador	0	1.572	No clear effect
Grenada	-0.037	1217.933	Negative: in Grenada, early reports are closer to final counts
Guadeloupe	-0.016	483.129	Negative: in Guadeloupe, early reports are closer to final counts
Guatemala	0	6.142	No clear effect
Guyana	-0.024	825.053	Negative: in Guyana, early reports are closer to final counts
Haiti	-0.001	17.578	Negative: in Haiti, early reports are closer to final counts
Honduras	0	1.053	No clear effect
Jamaica	-0.178	10408.081	Negative: in Jamaica, early reports are closer to final counts
Martinique	-0.02	703.53	Negative: in Martinique, early reports are closer to final counts
Mexico	0	0.396	No clear effect
Montserrat	-4.789	474057.126	Negative: in Montserrat, early reports are closer to final counts
Nicaragua	0	0.191	No clear effect
Panama	0	4.178	No clear effect
Peru	0	0.206	No clear effect
Puerto Rico	-0.015	399.901	Negative: in Puerto Rico, early reports are closer to final counts
Saint Barthelemy	-0.064	3096.266	Negative: in Saint Barthelemy, early reports are closer to final counts
Saint Kitts and Nevis	-10.095	2233849.075	Negative: in Saint Kitts and Nevis, early reports are closer to final counts
Saint Lucia	-1.585	133095.388	Negative: in Saint Lucia, early reports are closer to final counts
Saint Martin	-0.265	19061.499	Negative: in Saint Martin, early reports are closer

			to final counts
Saint Vincent and the Grenadines	-0.595	47203.666	Negative: in Saint Vincent and the Grenadines, early reports are closer to final counts
Suriname	-3.623	291941.418	Negative: in Suriname, early reports are closer to final counts
Trinidad and Tobago	-0.327	18371.704	Negative: in Trinidad and Tobago, early reports are closer to final counts
Turks and Caicos Islands	-2.999	1083253.969	Negative: in Turks and Caicos Islands, early reports are closer to final counts
United States of America	-0.011	317.541	Negative: in United States of America, early reports are closer to final counts
Uruguay	-0.201	11026.085	Negative: in Uruguay, early reports are closer to final counts
Venezuela	0	6.858	No clear effect
Virgin Islands (UK)	-0.505	36207.488	Negative: in Virgin Islands (UK), early reports are closer to final counts

Supp Table 3. Country coefficients of country model with a Generalised linear regression framework using a quasi-Poisson distribution (formula: Nts_V ~ Nts: (d + country)).
